# The Gilles de la Tourette Syndrome-Quality of Life Scale (GTS-QOL): A Validation in Japanese Patients

**DOI:** 10.3389/fpsyt.2021.797037

**Published:** 2022-01-03

**Authors:** Ryunosuke Goto, Natsumi Matsuda, Maiko Nonaka, Yu Hamamoto, Yosuke Eriguchi, Mayu Fujiwara, Akane Suzuki, Yukari Yokoyama, Yukiko Kano

**Affiliations:** ^1^Department of Pediatrics, The University of Tokyo Hospital, Tokyo, Japan; ^2^Department of Child Psychiatry, The University of Tokyo Hospital, Tokyo, Japan; ^3^Department of Developmental Psychology, Faculty of Human Studies, Shirayuri University, Tokyo, Japan; ^4^Graduate School of Education, The University of Tokyo, Tokyo, Japan; ^5^Department of Child Neuropsychiatry, Graduate School of Medicine, The University of Tokyo, Tokyo, Japan; ^6^Department of Social Welfare, Faculty of Social Welfare, Nihon Fukushi University, Mihama, Japan

**Keywords:** Tourette Syndrome, quality of life, tics, health-related quality of life, Gilles de la Tourette Syndrome, GTS-QOL—Gilles de la Tourette Syndrome–Quality of Life

## Abstract

**Background:** Though Gilles de la Tourette's syndrome (GTS) has significant impact on the quality of life of its patients, measures of health-related quality of life (HR-QOL) specific to adolescents and adults with GTS were not developed until recently. The present study provides evidence on the validity of the Gilles de la Tourette Syndrome-Quality of Life Scale (GTS-QOL), the first disease-specific HR-QOL instrument for GTS patients, for the first time in an East Asian sample.

**Methods:** One hundred and two Japanese individuals aged 13 and above with GTS were included in our study. Internal consistency was evaluated using Cronbach's alpha. The 4-factor structure of the GTS-QOL was assessed using confirmatory factor analysis, using goodness of fit indices, factor loadings of each questionnaire item, and covariances between factors. Validity was assessed using interscale correlations. Convergent and discriminate construct validity was evaluated using correlations with other scales such as the 28-item General Health Questionnaire, the Yale Global Tic Severity Scale, and the short version of the Padua Inventory.

**Results:** Scaling assumptions were met. Internal consistency reliability was high, with a Cronbach's alpha of 0.96. Confirmatory factor analysis revealed sufficient factor loadings and goodness of fit. All measures of goodness of fit corroborated the fit of the 4-factor model. Standardized covariances between factors in the confirmatory factor analysis were >0.8. There were significant correlations with other well-validated scales, and thus convergent and discriminate construct validity was sufficient.

**Conclusion:** The GTS-QOL is a valid and reliable instrument to measure disease-specific HR-QOL of GTS patients in Japan.

## Introduction

Gilles de la Tourette's Syndrome (GTS) is a neurological disorder characterized by multiple motor and vocal tics that often appears in childhood and affects up to 1% of school age children (1). In many medical conditions, quality of life (QOL) is increasingly being used as an important outcome measure for monitoring health conditions and effects of interventions (2), and GTS is no exception. Ever since the first study on the health-related quality of life (HR-QOL) of GTS patients (3), multiple studies have shown that patients with GTS have lower QOL than the general population (4). A similar study in Japan showed that tic severity was associated with lower HR-QOL (5). Furthermore, severe tics have been reported to result in pain and injuries (6), impairment in exercise and functional mobility has been shown to affect activities of daily living (7), and comorbidities like attention-deficit/hyperactivity disorder (ADHD) can affect school environments (8).

Despite the growing evidence of lower HR-QOL in patients with the disorder, a HR-QOL measure specific for GTS that addresses the various facets of the disorder that have significant impact on the patient's QOL was not introduced until recently (9). The first instrument to do so, the Gilles de la Tourette Syndrome-Quality of Life Scale (GTS-QOL), is a self-administered questionnaire that consists of 27 questions as well as a visual analog scale (GTS-QOL VAS) of how satisfied the patient feels with his/her life (9).

The GTS-QOL overcame the major shortcoming of applying generic HR-QOL instruments to HR-QOL of GTS patients; these instruments fail to address features specific to GTS patients that may cause distress, such as motor and vocal tics and repetitive behaviors (9). A previous study of young patients with GTS reported that generic HR-QOL was predicted primarily by comorbidities of obsessive-compulsive disorder (OCD) and attention deficit-hyperactivity disorder (ADHD) and not the severity of tics (10), though in reality many patients suffer from severe tics too. This further underscored the shortcomings of using generic HR-QOL measures, such as the Short Form Health Survey and Euro-Qol-5 Dimension in assessing HR-QOL in GTS patients (11). Furthermore, though clinical correlates in the QOL of GTS patients had been explored in previous studies, they relied on generic HR-QOL instruments (12, 13); usage of the GTS-QOL may allow researchers to better capture the different dimensions of the QOL of patients of a multifaceted disorder.

Particularly, though generic measures of HR-QOL have the advantage of allowing comparison across different diseases, they fail to address features unique to GTS, such as repetitive behaviors, motor and vocal tics, and coprolalia, as well as common comorbidities and problems in social life. On the other hand, assessment of disease status by clinical rating scales of tic severity alone omits the patient's subjective views about issues important to their lives, especially cognitive and socioemotional functioning. The development of the GTS-QOL was significant in that it conquered these shortcomings and provided clinicians and researchers with a tool to better characterize each GTS patient and monitor the clinical course or treatment effects over time.

Since its introduction in 2008, multiple versions of the GTS-QOL have been validated (14, 15) and multiple studies have utilized the instrument to measure HR-QOL in GTS patients (16–18). However, these studies were performed on Western populations, and no study has validated the GTS-QOL in East Asian populations. Though GTS has a biological origin, the expression of GTS symptoms and what symptoms patients might consider distressing may be influenced by cultural differences (19). It is thus important to assess whether the GTS-QOL is applicable in non-Western cultures. The present study is the first to validate the GTS-QOL in an East Asian sample.

## Materials and Methods

### Data Collection

#### Inclusion Criteria

We included patients with GTS who were aged 13 years or older who fully answered the GTS-QOL (including the GTS-QOL VAS).

#### Questionnaire Translation

The Japanese version of the GTS-QOL, including the GTS-QOL VAS, was translated and back-translated by an experienced GTS researcher.

#### Questionnaire Distribution and Interviews

The questionnaires were distributed to GTS patients who were members of the Tourette Syndrome Association of Japan (TSAJ, a non-profit organization for patients with GTS) and patients who attended the outpatient GTS clinic of the University of Tokyo Hospital or local psychiatry facilities in Tokyo and Kanagawa prefectures at the time of the study. The patients were asked to mail in or hand in the completed questionnaires. Additionally, outpatients of the University of Tokyo Hospital were asked to complete an interview by a psychologist for the assessment of the Yale Global Tic Severity Scale (YGTSS). Three hundred thirty-two patients were recruited from October 2019 to March 2020, and of these patients, we obtained informed consent from 95 of 112 (84.8%) patients from the University of Tokyo Hospital, 51 of 180 (28.3%) patients from TSAJ, and 28 of 40 (70.0%) patients from local psychiatry facilities in Tokyo or Kanagawa, making a total of 174 patients. Informed consent was obtained either by written consent after a participant received an explanation about the study (University of Tokyo Hospital Patients) or by answering “yes” to one of the questions in the questionnaire asking if the participant agreed to participate in the study (TSAJ or local psychiatry facilities).

### Instruments

In addition to the GTS-QOL, we included questions from the following scales in the questionnaire. We used 28-item General Health Questionnaire (GHQ-28) (20), a self-administered questionnaire whose reliability and validity have been investigated extensively both in Japan (21) and elsewhere (22, 23), as a screening tool for emotional distress. The GHQ-28 includes questions on somatic symptoms, anxiety and insomnia, social dysfunction, and severe depression (20). The severity of tics was assessed with the YGTSS, which assesses the severity and frequency of motor and phonic tics as well as how the tics impact the daily life and activities (24). Global functioning was assessed using the Global Assessment of Functioning (GAF) (25). Both YGTSS and GAF were performed by trained psychologists. The shortened version of the Padua Inventory (PI), whose reliability and validity has been reported in Japan, was used for assessing the severity of obsessive and compulsive symptoms, and includes questions on obsessions and compulsions regarding precision, impulses, contamination, checking, and rumination (26, 27).

### Statistical Analyses

#### Scaling Assumptions

We calculated the skewness and the ceiling and floor effects of the total GTS-QOL as well as the 4 subscales. We also calculated item mean scores, standard deviations, and item-total correlations.

#### Reliability

We calculated Cronbach's alpha to test the internal consistency reliability of the GTS-QOL.

#### Internal Construct Validity

We conducted a confirmatory factor analysis using the 4-factor model identified previously (1), in order to check if there are sufficient factor loadings and goodness of fit. Goodness of fit measures we used were Chi-squared *p*-value, comparative fit index (CFI), root mean square error of approximation (RMSEA), and standardized root mean square residual (SRMR). Additionally, we evaluated standardized covariances between factors as well as interscale correlations of the subscales.

#### Convergent and Discriminant Construct Validity

We then calculated correlations between the GTS-QOL total score and age, duration of disease, GTS-QOL VAS, as well as various well-validated scales that measure psychological well-being, tic severity, social functioning, and obsessive-compulsive symptoms (introduced in the Instruments section). The correlation between GTS-QOL and each scale was calculated using data from participants whose answers for each scale were fully available, and significance level was set at *P* = 0.001, after Bonferroni correction for multiple hypothesis tests (28).

#### Ethical Considerations and Statistical Software

The study was approved by the ethics committee of the University of Tokyo [IRB number: 10183-(4)]. All statistical analyses were conducted using R version 3.6.3.

#### Supplementary Analyses

Our study sample was comprised of participants 13 years or older, whereas the participants in the original GTS-QOL study were 16 years or older (9). In order to ensure that the GTS-QOL was valid among participants aged 16 years or older, we conducted the above analyses after excluding participants aged 13–15 years. The results are shown in the Supplementary Material.

## Results

102 patients met the inclusion criteria. 75 participants (73.5%) were males, and 27 (26.5%) were females. The average age of the participants was 24.7 years (range, 13–56 years). YGTSS and GAF were performed on a total of 40 patients. 29.4% of participants reported having comorbid OCD, and 21.6% reported having comorbid ADHD. The prevalence of these comorbidities were similar to what was reported in similar settings (9, 29). The basic characteristics of the study sample, including the prevalence of self-reported diagnosed comorbidities, are shown in Table 1.

**Table 1:** Basic characteristics of the study sample.

	**Study sample** **(***n*** = 102)**
Male gender (%)	73.5
Age (years)	24.7 [11.4]
Age at onset (years)	8.5 [6.5]
Age at diagnosis (years)	12.0 [9.7]
Disease duration (years)	16.2 [11.3]
GTS-QOL total score	65.0 [28.7]
GTS-QOL VAS	55.2 [28.1]
GHQ-28 total score	8.9 [7.5]
YGTSS motor tic severity	11.3 [7.1]
YGTSS vocal tic severity	9.7 [6.5]
YGTSS total tic severity (motor + vocal)	20.4 [12.1]
YGTSS impairment	21.4 [12.5]
YGTSS global severity score	39.6 [23.6]
GAF	61.9 [15.4]
PI (short version) total score	33.0 [29.5]
OCD (%)	29.4
ADHD (%)	21.6
Depression (%)	7.8
Anxiety disorder (including panic disorder and phobias) (%)	13.7

*Categorical variables are expressed as percentages, and numeric variables are expressed as mean [standard deviation]. GTS-QOL, Gilles de la Tourette Syndrome-Quality of Life Scale; VAS, visual analog scale; GHQ-28, 28-item General Health Questionnaire; YGTSS, Yale Global Tic Severity Scale; GAF, Global Assessment of Functioning; PI, Padua Inventory; OCD, obsessive-compulsive disorder; ADHD, attention-deficit/hyperactivity disorder*.

### Internal Construct Validity

Confirmatory factor analysis revealed sufficient factor loadings and goodness of fit. All but one of the GTS-QOL items had factor loadings >0.60 (Table 2). “Memory problems” and “losing important things” had loadings of 0.60 and 0.52 on the cognitive factor, respectively (Table 2). Interscale correlations of the subscales were 0.69–0.77 (Table 3). All measures of goodness of fit corroborated the fit of the 4-factor model (Chi-squared *p*-value 1.00, CFI 1.00, RMSEA 0.00, SRMR 0.08) (Table 4).

**Table 2:** Factor loadings of each item after confirmatory factor analysis.

	**Factor 1: Psychological**	**Factor 2: Physical/ADL**	**Factor 3: Obsessive-compulsive**	**Factor 4: Cognitive**
Depressed mood	0.85			
Lack of control over own life	0.86			
Loneliness/isolation	0.73			
Lack of self-confidence	0.80			
Frustration	0.80			
Lack of social support	0.79			
Anxiety	0.82			
Difficulty seeing friends	0.72			
Mood switches	0.79			
Temper dyscontrol	0.77			
Restlessness	0.84			
Embarrassing gestures		0.77		
Difficulty in daily life activities		0.70		
Involuntary swearing		0.66		
Pain or injuries		0.68		
Movement dyscontrol		0.67		
Phonic tics		0.72		
Difficulty taking part in social activities		0.72		
Repeating words			0.73	
Copying people			0.62	
Concerns about poor health			0.69	
Unpleasant thoughts			0.83	
Repeating actions			0.77	
Memory problems				0.60
Difficulty concentrating				0.86
Losing important things				0.52
Difficulty finishing tasks				0.65

*Factor loadings >0.4 were considered sufficient. ADL, activities of daily living*.

**Table 3:** Interscale correlations of each subscale.

	**Psychological subscale**	**Physical/ADL subscale**	**Obsessive-compulsive subscale**	**Cognitive subscale**
Psychological subscale	1.00			
Physical/ADL subscale	0.77	1.00		
Obsessive-compulsive subscale	0.77	0.77	1.00	
Cognitive subscale	0.73	0.69	0.74	1.00

*ADL, activities of daily living*.

**Table 4:** Scaling assumptions, reliability, and internal construct validity of the GTS-QOL and of each subscale.

	**GTS-QOL**	**Psychological subscale**	**Physical/ADL subscale**	**Obsessive-compulsive subscale**	**Cognitive subscale**
**Scaling assumptions**					
Range of item mean scores	1.83−2.97	2.11−−2.97	1.92−2.58	1.90−2.64	1.83−2.97
Range of item standard deviations	1.24−1.65	1.47−1.65	1.33−1.61	1.41−1.60	1.24−1.44
Range of corrected item-total correlations	0.48−0.82	0.62−0.84	0.54−0.72	0.55−0.74	0.44−0.64
Skewness	0.35	0.40	0.41	0.72	0.45
Floor/ceiling effects	0.06/0.01	0.11/0.02	0.17/0.02	0.21/0.03	0.16/0.01
**Reliability**					
Internal consistency (Cronbach's alpha)	0.96	0.95	0.87	0.86	0.75
**Goodness of fit**					
Chi-squared *p*-value	1.00				
CFI	1.00				
RMSEA	0.00				
SRMR	0.08				
**Correlation with other scales and individual characteristics**					
Age	0.11	0.05	0.17	0.20	0.06
Disease duration	0.11	0.04	0.18	0.13	0.03
GTS-QOL VAS	−0.60^***^	−0.65^***^	−0.47^***^	−0.46^***^	−0.46^***^
GHQ-28 total score	0.72^***^	0.78^***^	0.57^***^	0.60^***^	0.54^***^
YGTSS motor tic severity	0.35^*^	0.36^*^	0.38^*^	0.35^*^	0.14
YGTSS vocal tic severity	0.46^**^	0.38^*^	0.58^***^	0.42^**^	0.29
YGTSS total tic severity (motor + vocal)	0.55^***^	0.50^**^	0.64^***^	0.52^***^	0.34^*^
YGTSS impairment	0.65^***^	0.61^***^	0.66^***^	0.56^***^	0.49^**^
YGTSS global severity score	0.60^***^	0.56^***^	0.66^***^	0.52^***^	0.38^*^
GAF	−0.55^***^	−0.59^***^	−0.47^**^	−0.51^**^	−0.31
PI total score	0.77^***^	0.74^***^	0.63^***^	0.68^***^	0.65^***^

*For correlation coefficients with other scales*,

* *P < 0.05*,

** *P < 0.01, and*

*** *P < 0.001. GTS-QOL, Gilles de la Tourette Syndrome-Quality of Life Scale; ADL, activities of daily living; CFI, comparative fit index; RMSEA, root mean square error of approximation; SRMR, standardized root mean square residual; VAS, visual analog scale; GHQ-28, 28-item General Health Questionnaire; YGTSS, Yale Global Tic Severity Scale; GAF, Global Assessment of Functioning; PI, Padua Inventory*.

### Scaling Assumptions

All item response options were endorsed. Skewness of the total GTS-QOL was 0.35, and the skewness of the 4 subscales ranged from 0.40 to 0.72 (Table 4). The ceiling effect of the total GTS-QOL was 0.01 and the floor effect was 0.06. Ceiling effects of the 4 subscales ranged from 0.01 to 0.03 and floor effects from 0.11 to 0.21 (Table 4). Item mean scores and standard deviations were similar. Item-total correlations were >0.40 for all items (Table 4).

### Reliability

Internal consistency reliability was high, with a Cronbach's alpha of 0.96 (Table 4).

### Convergent and Discriminant Construct Validity

Convergent and discriminate construct validity was sufficient, with significant correlation between scales (GTS-QOL VAS −0.60, *P* < 0.001; GHQ-28 0.72, *P* < 0.001; YGTSS total tic severity 0.55, *P* < 0.001; GAF −0.55, *P* < 0.001; PI 0.77, *P* < 0.001; Table 4). The correlation between YGTSS vocal tic severity (0.46, *P* = 0.001) was larger than between YGTSS motor tic severity (0.35, *P* = 0.03). As for the subscales of GTS-QOL, the correlation between the psychological subscale and the GTS-QOL VAS (−0.65, *P* < 0.001) as well as GHQ-28 (0.78, *P* < 0.001) was the strongest of the four subscales. YGTSS total tic severity was most strongly correlated with the physical/ADL subscale of the GTS-QOL (0.64, *P* < 0.001). The PI total score was most strongly correlated with the psychological subscale (0.74, *P* < 0.001), followed by the obsessive-compulsive subscale (0.68, *P* < 0.001). Age and disease duration were not significantly correlated with GTS-QOL (0.11, *P* = 0.26; 0.11, *P* = 0.29). Other results of the correlations with scales are shown in Table 4.

## Discussion

### Principal Findings

The present study confirms the validity and reliability of the 27-item GTS-QOL in Japanese patients for the first time. As with other studies on the GTS-QOL, vocal tic severity was more strongly associated with GTS-QOL than motor tic severity, and measures of emotional distress (GHQ-28) and satisfaction with life (GTS-QOL VAS) were most strongly correlated with the psychological subscale (9, 14, 15). Furthermore, tic severity was most strongly correlated with the physical/ADL subscale of the GTS-QOL.

A study on Japanese GTS patients used both quantitative and qualitative methods to show that HR-QOL was lower than the general population and that tic severity and comorbid ADHD were associated with lower HR-QOL (5). This finding is corroborated by the confirmation of the Japanese GTS-QOL's cognitive subscale, which contains factors related to ADHD traits. The above study, from qualitative analysis of GTS patient interviews, also identified three main causes of distress in these patients, two of which appeared to be related to anxiety and depressive symptoms (anxiety about the future and unbearable emotional pain) (5). Our study, along with other studies which have utilized the GTS-QOL (9, 30), supports this finding, since these features are covered in the psychological subscale of the GTS-QOL, which was found to be most strongly associated with the GTS-QOL VAS. Hence, there seems to be a strong psychological component that determines the HR-QOL of GTS patients. We cannot conclude whether this phenomenon applies to GTS patients in any setting or is unique to Japanese patients, but psychological distress in GTS patients warrants further investigation.

Compared to the British study sample in the original GTS-QOL study by Cavanna et al., the mean GTS-QOL total score was greater in the present study's Japanese sample (65.0 in the present study compared to 35.9 in Cavanna et al.'s study), despite the fact that the mean YGTSS total tic severity was smaller in the present study (20.4 compared to 26.8). The Japanese sample may have been more prone to feel more distressed about their symptoms compared to the British sample, even if the severity of their tic symptoms were similar. This could be explained by the strong psychological component of HR-QOL of Japanese GTS patients described previously, though further research is necessary to confirm this. Importantly, GTS-QOL may not necessarily have cross-cultural equivalence, and cultural context should be taken into account when evaluating the total GTS-QOL score.

### Strengths and Limitations of This Study

Our study is of great significance in that studies on the HR-QOL, let alone disease-specific QOL, of GTS patients is very limited. Furthermore, other than the aforementioned mixed method study on the HR-QOL of Japanese GTS patients, studies on HR-QOL in GTS patients are rarely conducted in Japan, and the present study should open the door to more studies in this field.

Our study should be interpreted in light of several limitations. The patients were recruited at a tertiary referral hospital (the University of Tokyo Hospital) as well as a patient association, and this may have caused our sample from deviate away from the actual population of GTS patients in Japan. Furthermore, we were unable to perform test-retest reliability analysis since the questionnaire was administered only once to each patient, though our findings to support the reliability and validity of the Japanese version of the GTS-QOL, to the extent possible.

Additionally, our study sample consisted of participants 13 years or older, whereas the participants in the original GTS-QOL study were 16 years or older (9). Although there is a HR-QOL measure available for children and adolescents called the GTS-QOL for Children and Adolescents (C&A-GTS-QOL), few studies have validated this version compared to the GTS-QOL (14, 15), and we thus tested whether the GTS-QOL is valid even when we included individuals aged 13–15 years. This is a reasonable research question, as the C&A-GTS-QOL uses a self-report questionnaire for individuals aged 13 years or older, whereas individuals aged 6–12 years are evaluated based on interviews (14). Our study provides novel evidence that the GTS-QOL may be adaptable to the adolescent population.

### Implications and Future Studies

Notwithstanding the limitations, our study has multiple clinical implications and provides opportunities for future research. HR-QOL is important in measuring the impact of a chronic disease on the patient (31). This is especially relevant in GTS, a neurological disease with multiple comorbidities that can be a cause of social impairment and psychological distress. Reliable HR-QOL measures are both discriminative and evaluative (32). Discriminative instruments can differentiate between different patients; the GTS-QOL is thus likely to aid the clinician in effectively characterizing each patient and tailor the treatment plan to the individual. Additionally, researchers can use the GTS-QOL to characterize the HR-QOL of GTS patients in light of various clinical presentation of tics. For instance, one may utilize the questionnaire to investigate the relationship between premonitory urges, ability to suppress tics, and quality of life in GTS patients. Additionally, though characterizing the social impact of GTS on adults with comorbid OCD was said to be difficult (30), the physical/ADL subscale of the GTS-QOL, which is distinct from the obsessive-compulsive subscale, may be better able to capture this. Answering such questions will allow clinicians to better characterize each GTS patient and help provide tailored solutions to his/her specific needs.

An effective evaluative instrument can detect important changes, large and small, over a period of time (32). In this regard, the utility of the GTS-QOL should be confirmed in additional, longitudinal studies; one may wish to investigate the effects of treatments over time or monitor the clinical course of a GTS patient over multiple outpatient visits. For instance, utilizing the GTS-QOL, studies have found that in the QOL of adults with GTS, cognitive factors play a larger role compared to children (9, 14), which warrants further studies on cognitive function and GTS from early childhood to adulthood. Another study indicated that perceived impact of OCD on GTS-QOL seemed to decrease as the patients developed into adulthood (4, 18). Additional longitudinal studies will corroborate the utility of the GTS-QOL in monitoring patients over time.

As the discriminative and evaluative performance of the GTS-QOL is confirmed in additional studies, it can be used to collectively capture features of GTS other than tics, such as obsessive-compulsive symptoms, ADHD traits, depressive symptoms, and social functioning, over time. A study has shown that by early adulthood, tic symptoms will be greatly diminished in three-quarters of children with GTS and one-third will be tic-free (33). Hence, as the child grows into adolescence and adulthood it becomes increasingly important to capture the effects of problems in social life and comorbid conditions; for instance, comorbid conditions like OCD, depression, and anxiety disorders are more common in adolescence and early adulthood of GTS patients than the general population (33), all of which can be captured with the GTS-QOL's subscales. As shown above, the GTS-QOL is able to cover various aspects of GTS and can guide the clinician in effectively providing aid to a young GTS patient over time.

To date, the GTS-QOL (its child and adolescent variant) has only been applied to GTS patients as young as 7 years old (15). The onset of tics is typically 4–6 years old (34), and children as young as 4–6 years old with tics have been reported to exhibit greater distress when they exhibit comorbid compulsive-like behavior (35) [similar to older patients, as shown in the present study and in a previous study using a generic HR-QOL instrument on HR-QOL of GTS patients (10)]. Given its long clinical course, the GTS patients may benefit from the extension of the coverage of GTS-QOL to younger populations.

## Conclusion

The validity and reliability of the 27-item GTS-QOL was confirmed in Japanese GTS patients.

## Data Availability Statement

The datasets presented in this article are not readily available because the Ethics Review Board has not authorized data sharing of this research. Inquiries regarding the data should be directed to Yukiko Kano, kano-tky@umin.ac.jp.

## Ethics Statement

The studies involving human participants were reviewed and approved by the Ethics Committee of the University of Tokyo. Written informed consent to participate in this study was provided by the participants' legal guardian/next of kin.

## Author Contributions

RG, YY, and YK conceived the research idea. RG conducted the analyses and wrote the manuscript. YK directed the research. NM, MN, YH, YE, MF, AS, YY, and YK gave critical feedback on the research and approved the final manuscript. All authors contributed to the article and approved the submitted version.

## Funding

This work was supported by Health and Labor Sciences Research Grants for Comprehensive Research on Disability Health and Welfare from the Ministry of Health, Labor, and Welfare of Japan (Grant No. 19GC1001) and Grant-in-Aid for Scientific Research (C) (Grant No. 20K03435) from the Ministry of Education, Culture, Sports, Science and Technology in Japan.

## Conflict of Interest

The authors declare that the research was conducted in the absence of any commercial or financial relationships that could be construed as a potential conflict of interest.

## Publisher's Note

All claims expressed in this article are solely those of the authors and do not necessarily represent those of their affiliated organizations, or those of the publisher, the editors and the reviewers. Any product that may be evaluated in this article, or claim that may be made by its manufacturer, is not guaranteed or endorsed by the publisher.
