# Supplementary material for: The Gilles de la Tourette Syndrome-Quality of Life Scale (GTS-QOL): A Validation in Japanese Patients
Source: Front Psychiatry. 2022 Jan 3;12:797037. doi: 10.3389/fpsyt.2021.797037 (PMC8761978; doi:10.3389/fpsyt.2021.797037)
Supplement: Supplementary file 1 [file Table_1.PDF]

The Gilles de la Tourette Syndrome-Quality of Life Scale (GTS-QOL): a validation in Japanese patients

Ryunosuke Goto, MD; Natsumi Matsuda, PhD; Maiko Nonaka, PhD; Yu Hamamoto, MD; Yosuke Eriguchi, MD; PhD; Mayu Fujiwara, MA; Akane Suzuki, MA; Yukari Yokoyama, PhD; Yukiko Kano, MD, PhD

SUPPLEMENT

Results after excluding participants aged 13 to 15 years.

The validity and reliability statistics of the GTS-QOL after excluding participants aged 13 to 15 years are shown in Tables S1 to S3. 78 participants were aged 16 years or older (and 24 participants were aged 13 to 15 years). The results indicated good validity and reliability.

Table S1. Factor loadings of each item after confirmatory factor analysis.

	Factor 1: Psychological	Factor 2: Physical/ADL	Factor 3: Obsessive-compulsive	Factor 4: Cognitive
Depressed mood	0.85			
Lack of control over own life	0.90			
Loneliness/isolation	0.70			
Lack of self-confidence	0.79			
Frustration	0.82			
Lack of social support	0.77			
Anxiety	0.81			
Difficulty seeing friends	0.74			
Mood switches	0.79			
Temper dyscontrol	0.79			
Restlessness	0.82			
Embarrassing gestures		0.81		
Difficulty in daily life activities		0.69		
Involuntary swearing		0.70		
Pain or injuries		0.68		
Movement dyscontrol		0.65		
Phonic tics		0.70		
Difficulty taking part in social activities		0.70		
Repeating words			0.72	
Copying people			0.63	
Concerns about poor health			0.73	
Unpleasant thoughts			0.88	
Repeating actions			0.78	
Memory problems				0.65
Difficulty concentrating				0.87
Losing important things				0.54
Difficulty finishing tasks				0.73

Factor loadings>0.4 were considered sufficient. ADL, activities of daily living.

Table S2. Interscale correlations of each subscale.

	Psychological subscale	Physical/ADL subscale	Obsessive-compulsive subscale	Cognitive subscale
Psychological subscale	1.00			
Physical/ADL subscale	0.75	1.00		
Obsessive-compulsive subscale	0.79	0.81	1.00	
Cognitive subscale	0.73	0.71	0.76	1.00

ADL, activities of daily living.

Table S3. Scaling assumptions, reliability, and internal construct validity of the GTS-QOL and of each subscale.

	GTS-QOL	Psychological subscale	Physical/ADL subscale	Obsessive-compulsive subscale	Cognitive subscale
Scaling assumptions					
Range of item mean scores	1.89-3.08	2.22-3.08	2.01-2.64	2.09-2.69	1.89-2.94
Range of item standard deviations	1.26-1.65	1.47-1.65	1.34-1.64	1.45-1.60	1.26-1.48
Range of corrected item-total correlations	0.50-0.84	0.64-0.84	0.51-0.74	0.59-0.74	0.48-0.69
Skewness	0.27	0.35	0.34	0.57	0.40
Floor/ceiling effects	0.06/0.01	0.12/0.03	0.13/0.03	0.18/0.04	0.18/0.01
Reliability					
Internal consistency (Cronbach's alpha)	0.96	0.95	0.87	0.87	0.79
Goodness of fit					
Chi-squared p-value	1.00				
CFI	1.00				
RMSEA	0.00				
SRMR	0.09				
Correlation with other scales and individual characteristics					
Age	0.05	0.01	0.09	0.08	0.06
Disease duration	0.03	-0.03	0.12	0.03	0.00
GTS-QOL VAS	-0.54***	-0.61***	-0.39***	-0.44***	-0.46***
GHQ-28 total score	0.73***	0.80***	0.56***	0.58***	0.55***
YGTSS motor tic severity	0.36*	0.37*	0.41*	0.36*	0.13
YGTSS vocal tic severity	0.49**	0.39*	0.62***	0.45**	0.30
YGTSS total tic severity (motor + vocal)	0.50**	0.45**	0.62***	0.49**	0.28
YGTSS impairment	0.67***	0.61***	0.70***	0.57***	0.50**
YGTSS global severity score	0.59***	0.54***	0.66***	0.51**	0.35*
GAF	-0.51**	-0.54**	-0.43*	-0.47**	-0.24
PI total score	0.77***	0.74***	0.62***	0.67***	0.68***

For correlation coefficients with other scales, *P<0.05, **P<0.01, and ***P<0.001. GTS-QOL, Gilles de la Tourette Syndrome-Quality of Life Scale; ADL, activities of daily living; CFI, comparative fit index; RMSEA, root mean square error of approximation; SRMR, standardized root mean square residual; VAS, visual analogue scale; GHQ-28, 28-item General Health Questionnaire; YGTSS, Yale Global Tic Severity Scale; GAF, Global Assessment of Functioning; PI, Padua Inventory.
